# Buprenorphine Dispensing Following Medicaid Expansion Amid Unwinding in North Carolina

**DOI:** 10.1001/jamanetworkopen.2025.47933

**Published:** 2025-12-10

**Authors:** Joanne Constantin, Kao-Ping Chua, Jeffrey S. McCullough

**Affiliations:** 1 Susan B. Meister Child Health Evaluation and Research Center, Department of Pediatrics, University of Michigan Medical School, Ann Arbor; 2 Department of Health Policy and Management, School of Public Health, State University of New York (SUNY) Downstate Health Sciences University, Brooklyn; 3 Department of Health Management and Policy, University of Michigan School of Public Health, Ann Arbor

## Abstract

**Importance:**

The continuous Medicaid enrollment provision adopted during the COVID-19 pandemic ended in March 2023, after which 24 million US residents were disenrolled. Prior studies suggest this “unwinding” was associated with increased discontinuation of buprenorphine, an effective treatment for opioid use disorder. On December 1, 2023, North Carolina expanded Medicaid to low-income adults under the Patient Protection and Affordable Care Act (ACA).

**Objective:**

To evaluate changes in buprenorphine dispensing following North Carolina’s Medicaid expansion.

**Design, Setting, and Participants:**

This cross-sectional study used a difference-in-differences analysis of the IQVIA Longitudinal Prescription Database, which captures 92% of US prescriptions. The analysis was limited to North Carolina and South Carolina, which has not expanded Medicaid under the ACA. The sample included adults with Medicaid-paid buprenorphine prescriptions from March to May 2023, 3 months before unwinding began in these states. The preintervention period was June 1 to November 30, 2023, and the postintervention period was December 1, 2023 to December 31, 2024.

**Exposure:**

North Carolina’s Medicaid expansion.

**Main Outcomes and Measures:**

Monthly proportion of patients with at least 1 active buprenorphine prescription; monthly proportion of patients with at least 1 active prescription paid by Medicaid, private insurance, or cash. Linear probability models estimated differential changes in outcomes over time in North Carolina vs South Carolina.

**Results:**

Analyses included 286 216 person-months of data from 15 064 patients (mean [SD] age, 39.8 [9.2] years; 210 881 person-months [73.7%] contributed by women). The monthly proportion of patients with at least 1 active buprenorphine prescription declined by 11.3 and 11.2 percentage points in North Carolina and South Carolina, respectively, between June and November 2023. North Carolina’s Medicaid expansion was associated with a 1.6 (95% CI, 0.9-2.3) percentage point differential increase in this proportion, a 7.4 (95% CI, 5.7-9.1) percentage point differential increase in the probability of having at least 1 Medicaid-paid prescription, a −4.1 (95% CI, −5.2 to −3.0) percentage point differential change in the probability of having at least 1 private-pay prescription, and a −1.4 (95% CI, −2.6 to −0.3) percentage point differential change in the probability of having at least 1 cash-pay prescription.

**Conclusions and Relevance:**

In this cross-sectional study of prescription dispensing data, North Carolina’s Medicaid expansion was associated with a slowing of the decline in buprenorphine dispensing that occurred after unwinding of Medicaid continuous coverage requirement began. Medicaid expansion may have partially mitigated the adverse changes in buprenorphine dispensing associated with unwinding.

## Key Points

**Question:**

Among Medicaid-insured adults in North Carolina with buprenorphine dispensing before the end of Medicaid continuous coverage requirement in June 2023, was Medicaid expansion in December 2023 associated with changes in buprenorphine dispensing?

**Findings:**

In this cross-sectional study of prescription dispensing among 15 064 patients, buprenorphine dispensing decreased between June and November 2023 among individuals in North Carolina and South Carolina, which did not expand Medicaid. Medicaid expansion was associated with slowing of this decline in North Carolina compared with South Carolina.

**Meaning:**

The findings suggest that North Carolina’s Medicaid expansion may have partially mitigated the adverse changes in buprenorphine dispensing associated with the end of Medicaid continuous coverage requirement.

## Teaser

This cross-sectional study evaluates whether North Carolina’s Medicaid expansion was associated with changes in buprenorphine dispensing after the end of continuous Medicaid enrollment (unwinding) in March 2023.

## Introduction

The opioid epidemic has claimed more than 700 000 lives in the United States over the past 2 decades. In 2022 alone, more than 100 000 individuals died from drug overdoses, with opioids implicated in approximately 76% of these fatalities.^1^ Medicaid, the nation’s largest payer for individuals with opioid use disorder (OUD), plays a pivotal role in ensuring access to evidence-based treatment.^2^ This includes coverage for buprenorphine, a highly effective medication associated with increased treatment retention, reduced mortality, and improved rates of remission.^3,4,5,6,7,8,9^ Despite its vital role, access to buprenorphine remains precarious, particularly in the context of substantial Medicaid policy shifts over the past few years.

At the onset of the COVID-19 pandemic, Congress enacted the Families First Coronavirus Response Act, requiring states to maintain continuous Medicaid enrollment in exchange for enhanced federal funding support.^10^ Medicaid and Children’s Health Insurance Program enrollment thus surged by more than 23 million individuals by the end of March 2023.^10^ However, this continuous enrollment provision was decoupled from the public health emergency under the Consolidated Appropriations Act, formally expiring on March 31, 2023.^10^ This marked the beginning of the “Medicaid unwinding” process, during which more than 24 million individuals were disenrolled.^10^ Medicaid unwinding was associated with disruptions in buprenorphine therapy, raising concerns about the potential for increased opioid-related morbidity and mortality.^11^

Insurance coverage is closely tied to buprenorphine prescribing, positioning Medicaid as a critical tool in combating the opioid crisis.^12^ On December 1, 2023, North Carolina implemented Medicaid expansion under the Patient Protection and Affordable Care Act (ACA), extending eligibility to adults with incomes up to 138% of the federal poverty level.^13^ This policy rollout coincided with the state’s Medicaid unwinding process, creating a natural policy contrast. By April 2025, more than 650 000 individuals had enrolled through the expansion.^14^ This expansion presents a novel opportunity to assess whether expanding coverage can mitigate the adverse changes in buprenorphine dispensing associated with Medicaid unwinding. To our knowledge, aside from one simulation study,^15^ no empirical analysis has examined the association between North Carolina’s ACA Medicaid expansion and buprenorphine access. This study evaluates the association between this expansion and buprenorphine dispensing.

## Methods

### Data Source

For this cross-sectional study, buprenorphine dispensing data were derived from the IQVIA Longitudinal Prescription Database, an all-payer database that captures approximately 92% of prescriptions filled at US retail pharmacies. The database includes unique patient identifiers, enabling longitudinal tracking of prescription activity.^16^ Payment sources are classified as Medicaid, Medicare, cash, and commercial (including coupon, voucher, or discount card use). Information on state decisions to expand Medicaid under the ACA was obtained from the Kaiser Family Foundation.^17,18^ Because the data were deidentified, the University of Michigan Medical School Institutional Review Board exempted the study from human participant review, and informed consent was not required. This study follows the Strengthening the Reporting of Observational Studies in Epidemiology (STROBE) reporting guidelines for cross-sectional studies.^19^

### Study Design

We used a difference-in-differences design, leveraging state-level variation in the expansion of Medicaid presumptive eligibility during the Medicaid unwinding period. North Carolina served as the treatment state and neighboring South Carolina as the control state. The latter was selected given its geographic proximity and the fact that it had not expanded Medicaid under the ACA. Other potential control states—those that did not expand Medicaid under the ACA—were considered but ultimately excluded, as they did not satisfy the parallel trends assumption and therefore were not suitable as valid control groups (more details on the parallel trends assumption in the Statistical Analysis section). The preintervention period spanned June 1 to November 30, 2023, and the postintervention period extended from December 1, 2023, to December 31, 2024.

### Sample

The study sample included adults aged 18 to 64 years residing in North Carolina or South Carolina. These individuals must have had at least 1 active Medicaid-paid prescription for an immediate-release buprenorphine product during the baseline period. The baseline period was defined as the 3 months before the Medicaid unwinding process began (March to May 2023). Buprenorphine products approved for pain (eg, patches) were not included. A buprenorphine prescription was considered active from the dispensing date through the end date (dispensing date plus the days supplied minus 1). Individuals could have active buprenorphine prescriptions during the baseline period even if no buprenorphine was dispensed between March and May 2023, for instance, if a prescription filled in February had an active duration that extended into the baseline window. Each patient contributed 19 person-months of data, one for each month from June 2023 through December 2024. Person-months from adults who turned 65 years in either 2023 or 2024 were excluded due to potential eligibility for Medicare, which could offer prescription drug coverage to patients disenrolled from Medicaid during unwinding. Person-months from adults under the age of 65 years who were dually eligible for Medicaid and Medicare could not be excluded from the sample, as the database only reports the method of payment for prescriptions, which could differ from insurance type. However, fewer than 1% of person-months in the sample had active buprenorphine prescriptions paid with Medicare, suggesting the number of dually eligible adults under age 65 years was small.

### Outcomes

All outcomes were measured at the person-month level. The primary outcome was an indicator equal to 1 if a beneficiary had at least 1 active buprenorphine prescription, regardless of payment method. This outcome was a measure of treatment continuation. Three secondary outcomes included indicators for having at least 1 active buprenorphine prescription paid by Medicaid, private insurance, or self-pay. These outcomes captured potential changes in Medicaid coverage, transitions to private coverage, and transitions to uninsurance, respectively.

### Statistical Analysis

We used linear probability difference-in-differences models to evaluate whether changes in outcomes from the pre-expansion to postexpansion period differed between North Carolina and South Carolina. The models included an interaction term between indicators for North Carolina and the postexpansion period (the coefficient of interest), as well as 3-digit zip code fixed effects, month fixed effects, and variables for patient age in years and sex. Standard errors were clustered at the 3-digit zip code level. Results were virtually identical when using logistic regression models and calculating differences in average marginal effects at each level of the interaction term. eMethods 1 in Supplement 1 includes additional details on model specification.

The difference-in-differences framework rests on the assumption that, in the absence of North Carolina’s Medicaid expansion under the ACA, outcomes in North Carolina would have evolved in tandem with those in South Carolina. To assess the validity of this parallel trends assumption, we estimated event-study models incorporating month fixed effects (excluding November 2023 as the reference period), interactions between month and treatment group indicators, fixed effects for 3-digit zip code, and controls for patient age and sex. Statistical nonsignificance of pre-expansion interaction terms would support the parallel trends assumption. Further methodological details are provided in eMethods 2 in Supplement 1.

We stratified analyses based on patients’ buprenorphine treatment duration during the baseline period, comparing person-months from patients with at least 60 days vs less than 60 days of active buprenorphine prescriptions. The 60-day threshold was selected because some definitions of buprenorphine treatment retention permit up to a 30-day gap in medication supply.^20,21^

Statistical analyses were conducted using Stata, version 18.1/MP (StataCorp). Two-sided hypothesis tests were performed with a significance threshold of α = .05.

## Results

### Sample Characteristics

Table 1 displays the characteristics of the analytic sample overall and by group. The study included 286 216 person-months of data contributed by 15 064 unique patients (mean [SD] age, 39.8 [9.2] years), with 75 335 person-months (26.3%) from men and 210 881 (73.7%) from women. Of the total person-months, 226 252 (79.0%) were contributed by individuals residing in North Carolina, and 195 966 (68.5%) were from patients with at least 60 days of active buprenorphine coverage at baseline.

**Table 1.** Sample Characteristics

Characteristic	Person-months, No. (%)^a^		
	Total sample (N = 286 216)	North Carolina (n = 226 252 [79.0%])	South Carolina (n = 59 964 [21.0%]
Age, mean (SD), y	39.8 (9.2)	39.9 (9.3)	39.7 (8.9)
Age group, y			
18-25	10 155 (3.5)	8061 (3.6)	2094 (3.5)
26-34	76 258 (26.6)	61 359 (27.1)	14 899 (24.8)
35-44	126 432 (44.2)	97 597 (43.1)	28 835 (48.1)
45-54	47 196 (16.5)	38 016 (16.8)	9180 (15.3)
55-64	26 175 (9.1)	21 219 (9.4)	4956 (8.3)
Sex			
Male	75 335 (26.3)	61 294 (27.1)	14 041 (23.4)
Female	210 881 (73.7)	164 958 (72.9)	45 923 (76.6)

^a^ The sample was limited to person-months contributed by the cohort of adults with active buprenorphine prescriptions during the baseline period March to May 2023.

### Main Analysis

**Figure 1.**
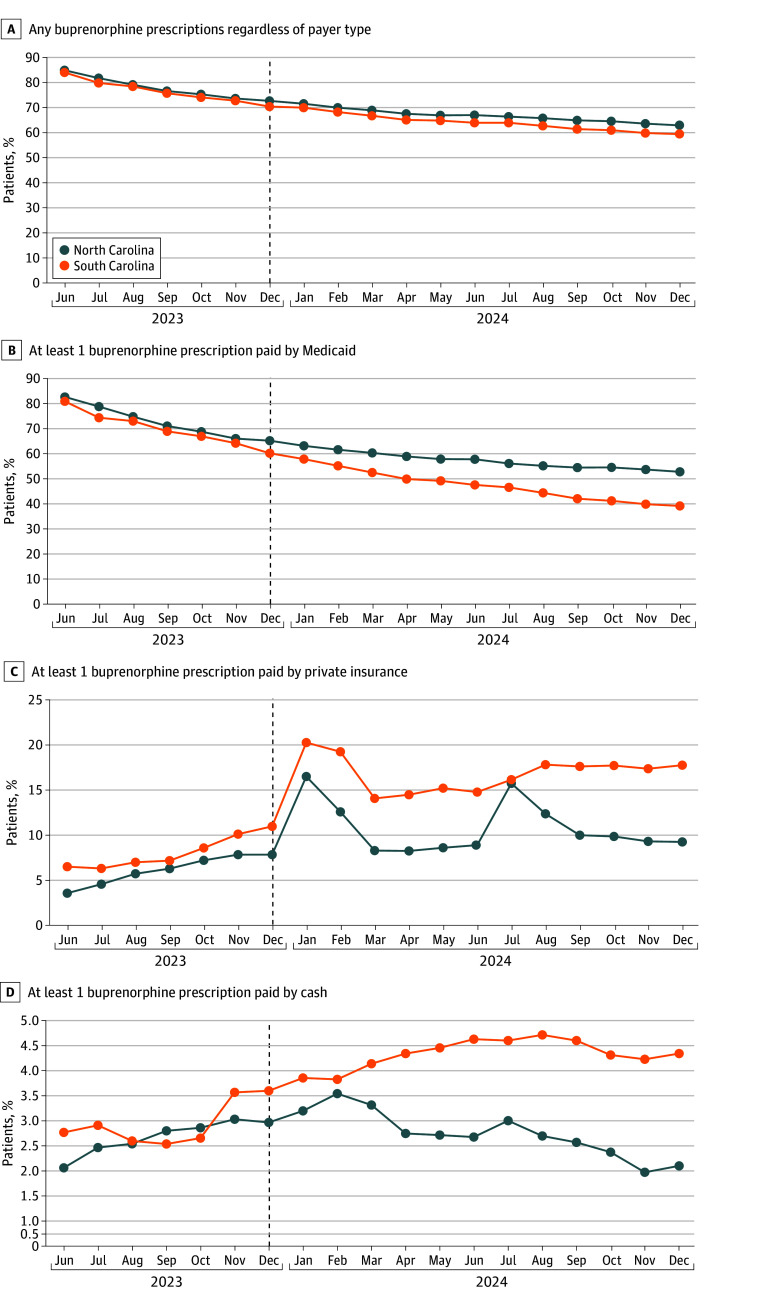
Trends in Unadjusted Outcomes in North Carolina and South Carolina, June 2023 to December 2024 The sample was limited to the cohort of adults with active buprenorphine prescriptions during the baseline period March to May 2023.

Figure 1 displays monthly trends in unadjusted outcomes by state. In both states, the proportion of patients with at least 1 active buprenorphine prescription declined between June and November 2023. In South Carolina, the proportion of individuals with at least 1 active prescription was 83.98% in June 2023 and 72.75% in November 2023 for a difference of 11.23 percentage points over this period; in North Carolina, the proportion of individuals with at least 1 active prescription was 84.89% in June 2023 and 73.6% in November 2023, for a difference of 11.29 percentage points over this period. Table 2 presents unadjusted outcomes for North Carolina and South Carolina during the preintervention and postintervention periods, along with difference-in-differences estimates. Among person-months from North Carolina, the proportion with at least 1 active buprenorphine prescription declined from 78.5% to 67.1% during the preintervention and postintervention periods. The corresponding decrease in South Carolia was 77. 4% to 64.4% (difference-in-differences estimate, 1.6 percentage points [95% CI, 0.9 to 2.3 percentage points]; *P* < .001).

**Table 2.** Difference-in-Differences Estimates of the Association Between Medicaid Expansion in North Carolina and Buprenorphine Dispensing

Outcome	Unadjusted outcome, No. (%)^a^				Adjusted difference-in-differences, estimate (95% CI), percentage points^b^
	North Carolina (treatment)		South Carolina (control)		
	Preintervention (n = 71 448)	Postintervention (n = 154 804)	Preintervention (n = 18 936)	Postintervention (n = 41 028)	
Primary outcome					
≥1 Active buprenorphine prescription	56 083 (78.5)	103 803 (67.1)	14 657 (77.4)	26 401 (64.4)	1.6 (0.9 to 2.3)^c^
Secondary outcomes					
≥1 Active buprenorphine prescription paid by Medicaid	52 621 (73.7)	89 438 (57.8)	13 518 (71.4)	19 727 (48.1)	7.4 (5.7 to 9.1)^c^
≥1 Active buprenorphine prescription paid by private insurance	4239 (5.9)	16 470 (10.6)	1454 (7.7)	6760 (16.5)	−4.1 (−5.2 to −3.0)^c^
≥1 Active buprenorphine prescription paid by cash	1700 (2.4)	3912 (2.5)	495 (2.6)	1727 (4.2)	−1.4 (−2.6 to −0.3)^d^

^a^ The sample was limited to the cohort of adults with active buprenorphine prescriptions during the baseline period of March to May 2023.

^b^ All estimates are at the person-month level. Each patient contributed up to 19 person-months between June 2023 and December 2024. The model included 3-digit zip code fixed effects, month fixed effects, and controlled for patient age in years and sex. Standard errors were clustered at the 3-digit zip code level.

^c^ *P* < .001.

^d^ *P* < .05.

**Figure 2.**
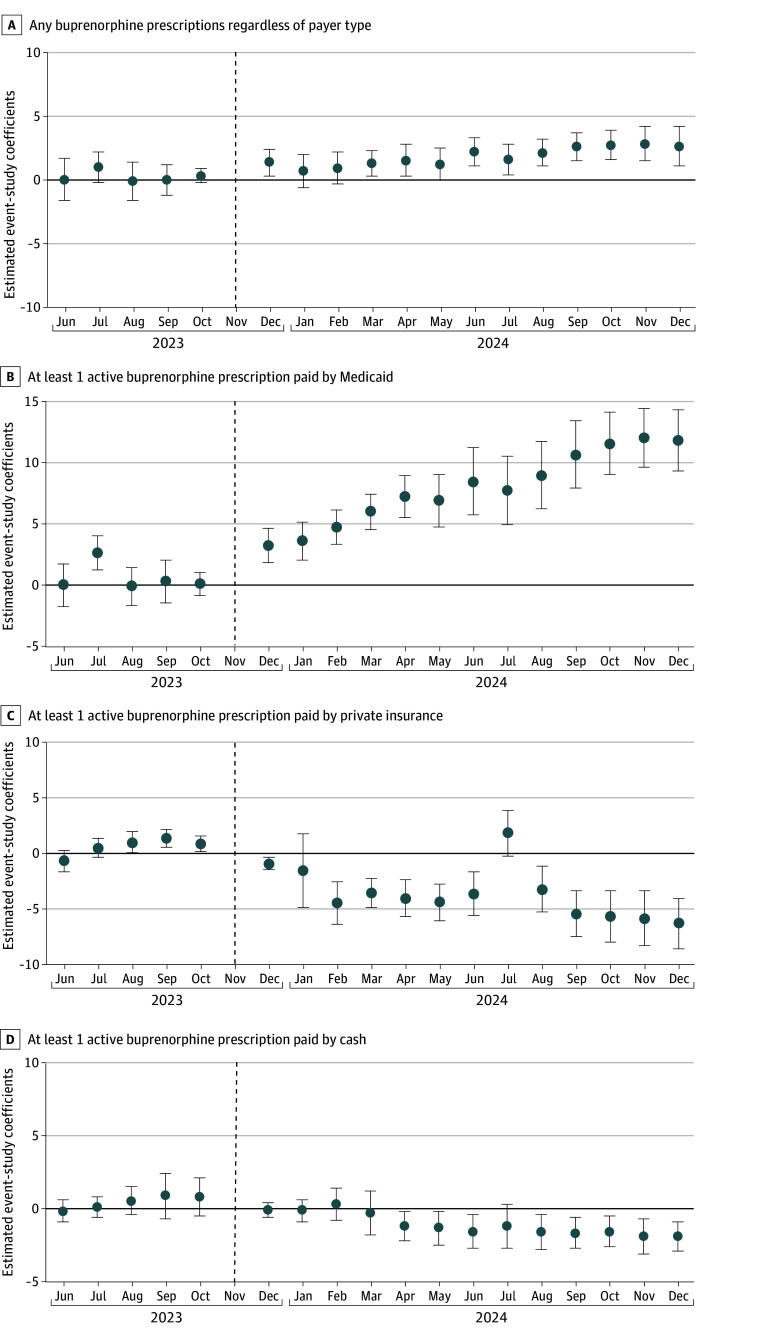
Event-Study Plots for the Association Between Medicaid Expansion in North Carolina and Buprenorphine Dispensing The sample is limited to the cohort of adults with active buprenorphine prescriptions during the baseline period March to May 2023. Whiskers indicate 95% CIs, and the dashed vertical line indicates the reference period (November 2023).

Among person-months from North Carolina, the proportion with any Medicaid-paid buprenorphine prescription decreased from 73.6% to 57.8% during the preintervention and postintervention periods, compared with a decrease from 71.4% to 48.1% in South Carolina (difference-in-differences estimate, 7.4 percentage points [95% CI, 5.7 to 9.1 percentage points]; *P* < .001). Conversely, the proportion of person-months with at least 1 active private insurance–paid buprenorphine prescription in North Carolina increased from 5.9% to 10.6% during the preintervention and postintervention periods, compared with 7.7% to 16.5% in South Carolina (difference-in-differences estimate, −4.1 percentage points [95% CI, −5.2 to −3.0 percentage points]; *P* < .001). The proportion of person-months with at least 1 cash-pay buprenorphine prescription in North Carolina increased from 2.4% to 2.5%, compared with 2.6% to 4.2% in South Carolina (difference-in-differences estimate, −1.4 percentage points [95% CI, −2.6 to −0.3 percentage points]; *P* = .02). Event study models supported the parallel trends assumption across outcomes, with minor violations observed in July 2023 (average marginal effect, [AME] 2.6 [95% CI, 1.4 to 4.0] percentage points) for Medicaid-pay prescriptions and August to October 2023 (eg, October 2023: AME, 0.9 [95% CI, 0.2 to 1.6] percentage points) for private-pay prescriptions (Figure 2).

### Subgroup Analyses

Table 3 presents the difference-in-differences estimates stratified by patients’ baseline buprenorphine treatment duration. Among person-months from patients with fewer than 60 days of active buprenorphine prescriptions at baseline, Medicaid expansion was not associated with a differential change in the probability of having at least 1 active buprenorphine prescription (−0.1 percentage points [95% CI, −1.7 to 1.5 percentage points]; *P* = .90), unlike person-months from patients with greater than 60 days of active buprenorphine prescriptions, in which Medicaid expansion was associated with a differential increase in this probability (2.5 percentage points [95% CI, 1.5 to 3.4 percentage points]; *P* < .001). Among the former group, expansion was associated with a smaller differential increase in the probability of having Medicaid-pay buprenorphine prescriptions (3.3 percentage points [95% CI, 1.0 to 5.7 percentage points]; *P* = .005) compared with the latter group (9.5 percentage points [95% CI, 7.7 to 11.3 percentage points]; *P* < .001). Expansion was also associated with a smaller differential decline in the probability of having private-pay buprenorphine prescriptions (−2.1 percentage points [95% CI, −4.1 to −0.04 percentage points]; *P* = .046) relative to the latter group (−5.1 percentage points [95% CI, −6.5 to −3.7 percentage points]; *P* < .001). Moreover, expansion was not associated with a differential change in the probability of having cash-pay prescriptions in the former group (−1.3 percentage points [95% CI, −2.9 to 2.6 percentage points]; *P* = .10) but was associated with a differential decrease in this probability in the latter group (−1.5 percentage points [95% CI, −2.6 to −0.4 percentage points]; *P* = .007).

**Table 3.** Difference-in-Differences Estimates of the Association Between Medicaid Expansion in North Carolina and Buprenorphine Dispensing: Subgroup Analyses

Outcomes	Adjusted difference-in-differences, by days with active buprenorphine prescriptions in baseline period, estimate (95% CI), percentage points^a^	
	<60 d (n = 90 250 person-months)	≥60 d (n = 195 966 person-months)
Primary outcome		
≥1 Active buprenorphine prescription	−0.1 (−1.7 to 1.5)	2.5 (1.5 to 3.4)^b^
Secondary outcomes		
≥1 Active buprenorphine prescription paid by Medicaid	3.3 (1.0 to 5.7)^b^	9.5 (7.7 to 11.3)^b^
≥1 Active buprenorphine prescription paid by private insurance	−2.1 (−4.1 to −0.04)^c^	−5.1 (−6.5 to −3.7)^b^
≥1 Active buprenorphine prescription paid by cash	−1.3 (−2.9 to 0.3)	−1.5 (−2.6 to −0.4)^b^

^a^ Treatment state is North Carolina, and control state is South Carolina. All estimates are at the person-month level. Each patient contributed up to 19 person-months between June 2023 and December 2024. The sample was limited to the cohort of adults with active buprenorphine prescriptions during the baseline period of March to May 2023. The model included 3-digit zip code fixed effects, month fixed effects, and controlled for patient age in years and sex. Standard errors were clustered at the 3-digit zip code level.

^b^ *P* < .001.

^c^ *P* < .05.

## Discussion

In this difference-in-differences study of adult Medicaid patients in North Carolina and South Carolina with buprenorphine dispensing, the proportion of patients with buprenorphine dispensing declined after Medicaid unwinding began in June 2023. North Carolina’s Medicaid expansion in December 2023 was associated with a slight slowing of this decline compared with the trend in South Carolina, which did not expand Medicaid. These findings suggest that North Carolina’s Medicaid expansion may have partially mitigated some of the adverse changes in buprenorphine dispensing that occurred after Medicaid unwinding began,^11^ thus helping preserve access to a key tool in efforts to slow the US opioid epidemic. It is also worth noting that other factors might have contributed to the broader national decline in buprenorphine dispensing prior to the expansion, such as shifts in treatment demand, changing overdose epidemiology,^1^ or potential increase in methadone use where access barriers eased.^22^

This study’s findings are consistent with studies that have shown that earlier Medicaid expansions under the ACA were associated with improved access to medications for OUD as well as with decreases in opioid-related mortality.^23,24,25^ The access improvements among the Medicaid population also outpaced improvements among patients with other health insurance whose treatment rates are still too low compared with rising OUD prevalence. Furthermore, during the peak of the COVID-19 pandemic, while overall health care utilization declined sharply, treatment for opioid overdoses rose substantially. This increase may reflect the federal continuous Medicaid enrollment requirement during that period, which helped sustain buprenorphine access among adult Medicaid beneficiaries. Together, these patterns underscore the critical role of Medicaid in ensuring access to buprenorphine treatment. Our study is unique in the Medicaid expansion literature in that the expansion in question occurred in the context of a rapid period of Medicaid disenrollment. Among adults with active buprenorphine prescriptions in March to May 2023, buprenorphine dispensing declined in both North Carolina and South Carolina, although the decline occurred more gradually in North Carolina following expansion. Thus, the expansion may have buffered against a steeper erosion in buprenorphine dispensing during unwinding.

In the Medicaid expansion literature, a key policy concern has been the notion of “crowd-out,” in which increases in Medicaid coverage are offset by decreases in private insurance coverage, mitigating or even nullifying net gains in overall insurance coverage.^26,27,28^ In the current study, there was a decrease in Medicaid-pay prescriptions and an increase in private-pay prescriptions in both North Carolina and South Carolina before December 2023. This pattern is consistent with a potential shift from Medicaid to private insurance, one that might be thought of as reverse crowd-out. Medicaid expansion was associated with a slowing in the decrease in Medicaid-pay prescriptions in North Carolina compared with South Carolina and with a slowing in the increase in private-pay prescriptions, suggesting that expansion was associated with a reduction in this reverse crowd-out phenomenon. This could be viewed as a favorable finding, as it suggests that expansion may have reduced transitions to costlier or less stable payment sources, such as commercial insurance.

In North Carolina, cash-pay prescriptions did not become meaningfully more common after December 2023, unlike in South Carolina. This finding suggests that Medicaid expansion in North Carolina may have helped limit transitions to uninsurance, consistent with prior literature. This reduced transition to uninsurance among individuals with OUD is important given the substantial out-of-pocket costs associated with OUD treatment.^29,30,31^ Thus, for some patients, North Carolina’s Medicaid expansion may have prevented the cost-related barriers to accessing buprenorphine that may otherwise have occurred due to loss of insurance coverage.

Among patients with at least 60 days of baseline buprenorphine use—those more likely to be stably engaged in treatment—North Carolina’s Medicaid expansion was associated with a slowing of the decrease in buprenorphine discontinuation and a slowing of the decrease in the use of Medicaid to pay for buprenorphine prescriptions. However, the same was not observed among patients with fewer than 60 days of baseline buprenorphine use. Findings suggest that the benefits of Medicaid expansion for buprenorphine dispensing may have been concentrated among patients with more established use. For those with less established use, Medicaid expansion alone was insufficient to mitigate the adverse changes in buprenorphine dispensing associated with Medicaid unwinding.

It is worth noting that the differential changes associated with North Carolina’s Medicaid expansion were relatively modest in magnitude. This likely stems, in part, from the inclusion in the analytic sample of individuals who both did and did not regain Medicaid coverage following disenrollment during unwinding. It is possible that the observed associations would have been more substantial if data had permitted restricting the sample to individuals who successfully reenrolled.

Even with this caveat in mind, however, the modest magnitudes of the reported estimates suggest that any efforts by nonexpansion states to expand Medicaid in the future would likely be insufficient to fully mitigate the adverse changes in buprenorphine dispensing associated with Medicaid unwinding. Policymakers in all states, regardless of whether they have expanded Medicaid or not, should invest in other strategies to preserve access to buprenorphine among Medicaid patients who were disenrolled due to unwinding. These strategies might include targeted outreach to Medicaid-eligible individuals who were erroneously disenrolled due to procedural issues, streamlining administrative processes by simplifying and automating reenrollment and eligibility verification, strengthening coordination between state agencies and health care practitioners, and connecting beneficiaries to community-based treatment resources, such as local opioid treatment centers, harm reduction services, and peer support networks. If combined with Medicaid expansion, these strategies could help ensure sustained access to buprenorphine during a critical time in the US opioid epidemic.

### Limitations

This study has limitations. First, data limitations precluded the ability to examine whether Medicaid expansion was associated with changes in opioid-related adverse events, such as overdose. Second, estimates may be biased if other state-level policies, such as changes to advanced practitioner practice scope or the nature of buprenorphine prior authorization requirements, differentially impacted dispensing following expansion; however, we are unaware of any such concurrent policy changes. Third, there were some violations of the parallel trends assumption, although the magnitude of these violations was small. Fourth, because the database lacked information on whether a given patient gained Medicaid coverage due to expansion, we could not estimate the association between gaining coverage and buprenorphine dispensing at the individual level. Estimates should rather be interpreted as the association between a state-level policy (ie, Medicaid expansion) and buprenorphine dispensing. Fifth, our analyses are limited to adults with an active Medicaid-paid buprenorphine prescription during March to May 2023 and do not capture patients initiating buprenorphine therapy after these months. Additionally, we were unable to ascertain whether patients relocated to another state or died. Any differential changes in either of these events between North and South Carolina following Medicaid expansion could introduce bias into our estimates.

## Conclusions

In this cross-sectional study, North Carolina’s Medicaid expansion was associated with a slight slowing of the decline in buprenorphine dispensing that occurred after Medicaid unwinding began. Future research should evaluate the association between North Carolina’s Medicaid expansion and adverse health outcomes, such as opioid overdose. Additionally, given that the magnitude of the changes in buprenorphine dispensing associated with Medicaid expansion were modest, future research should identify what other interventions can more effectively mitigate the adverse changes in buprenorphine dispensing associated with Medicaid unwinding.

## Notes

Supplement 1. **eMethods 1.** Details on the Difference-in-Differences Model **eMethods 2.** Details on the Event-Study Model

Supplement 2.
Data Sharing Statement
