# Supplementary material for: Buprenorphine Dispensing Following Medicaid Expansion Amid Unwinding in North Carolina
Source: JAMA Netw Open. 2025 Dec 10;8(12):e2547933. doi: 10.1001/jamanetworkopen.2025.47933 (PMC12696594; doi:10.1001/jamanetworkopen.2025.47933)
Supplement: Supplement 1. — eMethods 1. Details on the Difference-in-Differences Model eMethods 2. Details on the Event-Study Model [file jamanetwopen-e2547933-s001.pdf]

Supplemental Online Content

Constantin J, Chua KP, McCullough JS. Buprenorphine dispensing following medicaid expansion amid unwinding in North Carolina. *JAMA Netw Open*. 2025;8(12):e2547933. doi:10.1001/jamanetworkopen.2025.47933

eMethods 1. Details on the Difference-in-Differences Model

eMethods 2. Details on the Event-Study Model

This supplemental material has been provided by the authors to give readers additional information about their work.

© 2025 Constantin J et al. *JAMA Network Open*.

eMethods 1. Details on the Difference-in-Differences Model

The following regression model was estimated:

$$Y_{ist} = \beta_0 + \beta_1 * Expansion\ State_S * Post_t + X_{ist}\gamma + \tau_t + \sigma_s + \epsilon_{ist} \quad (1)$$

Y is a buprenorphine dispensing outcome for patient *i* in state *s* at month *t*. *Expansion_State* is a binary indicator that equals 1 for patients in North Carolina and 0 for patients in South Carolina. *Post* is a binary indicator that equals 1 for months between December 2023 and December 2024 and 0 for months between June and November 2023. Thus, the interaction term only equals 1 if data derive from North Carolina in any month starting December 2023 onward. The main difference-in-differences parameter is β_1, representing any differential change in outcomes between the pre-expansion period and the post-expansion period among patients in North Carolina versus South Carolina. **X** is a vector of patient characteristics that includes age and sex. $\boldsymbol{\tau}$ is a vector of month fixed effects, $\boldsymbol{\sigma}$ is a vector of zip code fixed effects, and ε is an idiosyncratic error term.

© 2025 Constantin J et al. *JAMA Network Open*.

eMethods 2. Details on the Event-Study Model

The following regression model was estimated:

$$Y_{ist} = \beta_0 + \sum(\beta_t * Expansion\ State_s * Month_t) + X_{ist}\gamma + \tau_t + \sigma_s + \epsilon_{ist} \ (2)$$

Similar to model (1), *Y* is one of the buprenorphine dispensing outcomes for patient *i* in state *s* in month *t*. *Expansion State* is a binary indicator that equals 1 for patients in North Carolina and 0 for patients in South Carolina. *Month* includes binary (0/1) indicators for each month from June 2023 to December 2024; November 2023 was the reference year. β_1–β_5 represent differences in changes from June 2023 through October 2023 relative to November 2023 between patients in North Carolina and South Carolina. If these coefficients are significant, this would suggest differential pre-expansion trends. **X** is a vector of patient characteristics that includes age and sex. $\boldsymbol{\tau}$ is a vector of month fixed effects, $\boldsymbol{\sigma}$ is a vector of zip code fixed effects, and ε is an idiosyncratic error term.

© 2025 Constantin J et al. *JAMA Network Open*.
