# Supplementary material for: Buprenorphine Dispensing Following Medicaid Expansion Amid Unwinding in North Carolina
Source: JAMA Netw Open. 2025 Dec 10;8(12):e2547933. doi: 10.1001/jamanetworkopen.2025.47933 (PMC12696594; doi:10.1001/jamanetworkopen.2025.47933)
Supplement: Supplement 2. — Data Sharing Statement [file jamanetwopen-e2547933-s002.pdf]

Data Sharing Statement

Constantin. Buprenorphine Dispensing Following Medicaid Expansion Amid Unwinding in North Carolina. *JAMA Netw Open*. Published December 10, 2025. doi:10.1001/jamanetworkopen.2025.47933

Data
Data available: No

Additional Information
Explanation for why data not available: IQVIA data is proprietary and cannot be shared.
